# Genotyping and Whole-Genome Sequencing to Identify Tuberculosis Transmission to Pediatric Patients in British Columbia, Canada, 2005–2014

**DOI:** 10.1093/infdis/jiy278

**Published:** 2018-05-11

**Authors:** Jennifer L Guthrie, Andy Delli Pizzi, David Roth, Clare Kong, Danielle Jorgensen, Mabel Rodrigues, Patrick Tang, Victoria J Cook, James Johnston, Jennifer L Gardy

**Affiliations:** 1 School of Population and Public Health, University of British Columbia, Vancouver, Canada; 2 Public Health and Preventive Medicine Residency Program, Cumming School of Medicine, University of Calgary, Alberta; 3 British Columbia Centre for Disease Control, Vancouver, Canada; 4 British Columbia Centre for Disease Control Public Health Laboratory, Vancouver, Canada; 5 Department of Pathology, Sidra Medical and Research Center, Doha, Qatar; 6 Department of Medicine, University of British Columbia, Vancouver, Canada

**Keywords:** genomic epidemiology, pediatric, transmission, tuberculosis

## Abstract

**Background:**

Tuberculosis (TB) in children is often an indicator of recent transmission. Genotyping and whole-genome sequencing (WGS) can enhance pediatric TB investigations by confirming or refuting transmission events.

**Methods:**

*Mycobacterium tuberculosis* isolates from all pediatric patients <18 years with culture-confirmed TB in British Columbia (BC) from 2005 to 2014 (*n* = 49) were genotyped by Mycobacterial Interspersed Repetitive Units–Variable Number Tandem Repeat (MIRU-VNTR) and compared with adult isolates. Genotypically clustered cases underwent WGS. Clinical, demographic, and contact data were reviewed for each case.

**Results:**

Twenty-three children were Canadian-born, 7 to Canadian-born parents (CBP) and 16 to foreign-born parents (FBP). Of the 26 foreign-born children, all were born in Asia (81%) or Africa (19%). Using molecular and epidemiological data, we determined that 15 children had acquired their infection within BC, and household transmission explained all 7 Canadian-born (FBP) children that acquired TB locally. In contrast, 6 of 7 Canadian-born (CBP) children were exposed via a non-household community source. Eight Canadian-born (FBP) children acquired their infections through travel to their parents’ place of birth. All but 1 of the foreign-born children acquired their infection outside of BC.

**Conclusions:**

Genotyping and genomic data reveal that drivers of pediatric transmission vary according to a child’s age, birthplace, and their parents’ place of birth.

## Teaser

Retrospective genotyping and whole-genome sequencing over a 10-year period suggested that pediatric tuberculosis in British Columbia is a mosaic and factors including age, birthplace, and travel history must all be considered together when inferring a pediatric patient’s likely exposure.

In 2016, there were an estimated 1 million new cases of childhood tuberculosis (TB), causing 253000 deaths globally [1]. In low-incidence countries such as Canada, children <18 years have the lowest TB rates of any age group [2]; however, pediatric cases are often difficult to diagnose and complex to manage. Presentations may be atypical, diagnostic yields are low, and pediatric patients are at greater risk of developing severe, disseminated, and potentially fatal disease without prompt treatment [3, 4]. Tuberculosis in young children is considered indicative of recent transmission. In high-resource regions, reverse contact tracing is usually performed in an attempt to identify and treat the source case to prevent further spread [5].

Contact investigations initiated around pediatric TB patients are most likely to identify a source case in children <2 years, typically revealing an adult caregiver or household member [5–7]. However, the epidemiology is not always clear. In a setting such as Canada, foreign-born children are less likely to be epidemiologically linked to a known case, and they are often presumed to have acquired infection before immigration [5, 8, 9]. Even when a putative source is identified, the molecular epidemiology may not always support a relationship between the *Mycobacterium tuberculosis* (*Mtb*) isolates of the child and presumed source. Genotyping has revealed instances in which a pediatric patient’s *Mtb* isolate has a different genotype from their assumed source case, thus refuting transmission [5, 10, 11], and discordant drug-susceptibility patterns have also been used to disprove transmission. Multidrug-resistant (MDR)-TB, particularly in children of immigrants, is believed to result from exposure to adult family members with MDR-TB [12, 13], yet at least 2 studies have shown conflicting resistance phenotypes between pediatric cases and their presumed source [11, 14].

In contrast, concordant genotypes and susceptibility patterns do not necessarily mean that a specific individual was the source of a child’s TB infection. Genotyping methods such as Mycobacterial Interspersed Repetitive Units–Variable Number Tandem Repeat (MIRU-VNTR) overestimate clustering in certain lineages of TB that are common among individuals born in high-incidence countries, thus identical genotypes in low-incidence countries often suggest infection with a strain common to a particular ethnic community [15, 16] rather than recent person-to-person transmission—a finding that might influence public health follow-up. The linkages between cases can be further refined through whole-genome sequencing (WGS), which—when combined with epidemiological data—can better identify transmission chains [17]. Using WGS, *Mtb* isolates with identical genotypes may be separated by enough genomic distance (>5 mutations) to rule out recent transmission from a putative source [18].

To better understand the transmission dynamics of pediatric TB in a low-incidence setting, we carried out a retrospective analysis of all culture-positive TB cases in children <18 years diagnosed in British Columbia (BC), Canada from 2005 through 2014. We used routine programmatic contact investigation data combined with MIRU-VNTR and WGS to identify source cases and to quantify the extent to which transmission within the province contributes to the overall burden of pediatric TB in BC.

## METHODS

### Study Setting and Design

The British Columbia Centre for Disease Control (BCCDC)’s Public Health Laboratory (BCPHL) receives all *Mtb* cultures for the province and performs routine phenotypic drug-susceptibility testing and MIRU-VNTR genotyping on all *Mtb* isolates. Patient care, surveillance, and TB prevention programs are led by Provincial TB Services at the BCCDC. We identified all children <18 years diagnosed with TB from 2005 through 2014 (*n* = 98) in BC from the provincial surveillance registry, and we restricted the study population to only those with a culture-positive diagnosis made in BC (*n* = 49).

### Case Data

Individual-level clinical, demographic, and contact investigation data were obtained for all study participants through BCCDC’s Integrated Provincial Health Information System (iPHIS). Disease site was categorized as respiratory or nonrespiratory [19]. Information regarding each child’s parents’ country of origin was obtained through a combination of physician narrative and contact investigation records in iPHIS. Children were categorized by birthplace as foreign-born or Canadian-born; the latter group was further subdivided into children born to foreign-born parents (FBP) or to Canadian-born parents (CBP). Ethnic community was defined by a foreign-born patient’s country of birth, or, for Canadian-born patients, their parents’ country of birth. In the one case in which the parents were from different countries, we used the ethnic community of the parent born in the higher TB incidence country (≥30 cases/100000) [19]. The University of British Columbia’s biosafety and ethics committees reviewed and approved the study (certificate no. H12-00910).

### Laboratory Methods

*Mycobacterium tuberculosis* isolates were obtained from specimens submitted to the BCPHL for routine testing. Isolates were revived from archived stocks, deoxyribonucleic acid extracted, and genotyped using 24-locus MIRU-VNTR genotyping as previously described [20]. All 49 (100%) culture-positive isolates were successfully genotyped, and isolates whose MIRU-VNTR genotype matched 1 or more patient isolates in BC during the study period (see [20]) were assigned a cluster identifier (MClustID). All clustered isolates—those from the pediatric patients (*n* = 24) and all isolates from adult cases in each cluster (*n* = 202)—were sequenced using 125 base pairs, paired-end reads on the Illumina HiSeqX platform at the Michael Smith Genome Sciences Centre (Vancouver, BC).

### Whole-Genome Sequencing Analysis

The resulting fastq files were analyzed using a pipeline developed by Oxford University and Public Health England [21]. Reads were aligned to the *Mtb* H37Rv reference genome (GenBank ID: NC000962.2), with an average of 92% of the reference genome covered. Single-nucleotide variants (SNVs) were identified across all mapped nonrepetitive sites. Concatenated SNVs were used to construct maximum-likelihood phylogenetic trees (RAxML 8.2.10 [22]; GTRGAMMA model and 200 bootstrap replicates), which were then viewed using the R statistical software (version 3.4.1). Lineage-defining SNVs [23] were used to classify each sequenced isolate into 1 of the 7 genetic *Mtb* lineages. Fastq files for all genomes are available at the National Center for Biotechnology Information under BioProject PRJNA413593 and PRJNA49659.

### Transmission Classification

We combined WGS data with patient-level clinical and epidemiological data, including symptom onset and diagnosis dates, disease site(s), and contact investigation information, to identify the most probable source for each infection. Locally acquired infections were defined as follows: (1) those pediatric cases whose *Mtb* isolate fell within 0–5 SNVs of another isolate from someone diagnosed in BC and for which there was epidemiological support, or (2) those pediatric cases without WGS data but for which there was irrefutable epidemiological evidence of transmission. We defined TB infections acquired outside BC as those pediatric cases whose *Mtb* isolates had either a unique MIRU-VNTR pattern or who, by WGS, were >5 SNVs away from another isolate in BC and who had a documented history of residing in or traveling to a high-incidence TB country. Although other sources for these cases are possible—for example, a clinically diagnosed case unknown to the child, an adult source infecting a child before the study initiation, an unidentified visitor from outside BC, an individual whose active, infectious TB disease spontaneously resolved, or a case that left the province before diagnosis—these scenarios are considerably less likely. Pediatric patients not meeting either definition for place of acquisition were classified as having an unknown source.

### Statistical Analysis

We computed descriptive statistics for demographic, clinical, and contact investigation data, both overall and stratified by patient birthplace. Unadjusted differences in characteristics between birthplaces were analyzed using the Fisher’s exact test (categorical data) or the Kruskal-Wallis rank-sum test (nonnormal continuous data). In addition, we performed univariable analysis for factors related to locally acquired infection (yes/no) using the χ^2^ test or Fisher’s exact test, where appropriate. All analyses were executed in R (version 3.3.1).

## RESULTS

### Demographics, Clinical Presentation, and Epidemiology

**Figure 1.**
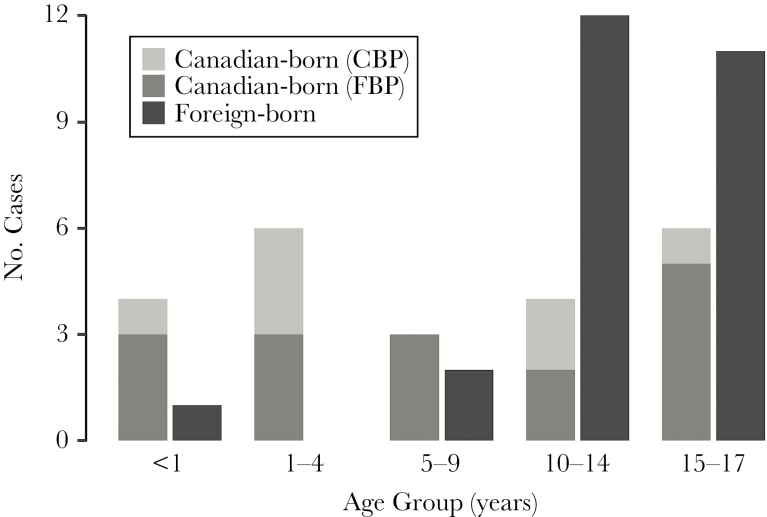
Pediatric age distribution by birthplace, British Columbia, 2005–2014. Abbreviations: Canadian-born parents (CBP); Foreign-born parents (FBP).

From 2005 to 2014, a total of 98 children were diagnosed with active TB in British Columbia; 49 (50.0%) children had at least 1 culture-positive isolate available at the BCPHL (Supplementary Figure 1). The median age of our study population was 14 (interquartile range [IQR], 6–16); however, the age distribution varied significantly between Canadian-born and foreign-born children (Table 1, Figure 1), with foreign-born children almost always >10 years of age.

**Table 1.** Demographic and Clinical Characteristics of Culture-Positive Pediatric TB Patients, British Columbia 2005**–**2014 (*n* = 49)^a^

Characteristic	Overall	Canadian-Born (CBP)	Canadian-Born (FBP)	Foreign-Born	*P* Value^b^
Totals	*n* = 49	*n* = 7	*n* = 16	*n* = 26	
Age, years					
Median (IQR)	14 (6–16)	4 (1–13)	7 (1–16)	15 (13–17)	.023
Gender—*n* (%)					
Male	25 (51.0)	2 (8.0)	10 (40.0)	13 (52.0)	.329
Female	24 (49.0)	5 (20.8)	6 (25.0)	13 (54.2)	
Ethnic community^c^—*n* (%)					
Multigenerational Canadian	7 (14.3)	7 (100.0)	–	–	–
South-Eastern Asia	21 (42.9)	–	7 (33.3)	14 (66.7)	
South-Central Asia	9 (18.4)	–	4 (44.4)	5 (55.6)	
East Asia	5 (10.2)	–	3 (60.0)	2 (40.0)	
Africa	7 (14.3)	–	2 (28.6)	5 (71.4)	
Disease Site—*n* (%)					
Respiratory	29 (59.2)	3 (10.3)	10 (34.5)	16 (55.2)	.471
Nonrespiratory	11 (22.4)	1 (9.1)	3 (27.3)	7 (63.6)	
Respiratory + Nonrespiratory	9 (18.4)	3 (33.3)	3 (33.3)	3 (33.3)	
Respiratory^d^ Smear—*n* (%)					
Positive	21 (53.8)	4 (19.0)	7 (33.3)	10 (47.6)	1.000
Cavitary					
Yes	4 (8.2)	1 (25.0)	1 (25.0)	2 (50.0)	.620
No. of Contacts					
Median (IQR)	5 (2–19)	17 (2–29)	5 (2–13)	5 (1–19)	.819
Method of Detection— *n* (%)					
Symptoms	39 (79.6)	5 (12.8)	12 (30.8)	22 (56.4)	.031
Contact Investigation	6 (12.2)	2 (33.3)	4 (66.7)	0 (0.0)	
Postlanding Surveillance	3 (6.1)	–	–	3 (100.0)	
Incidental Finding	1 (2.0)	1 (100.0)	0 (0.0)	0 (0.0)	
Clustered^e^—*n* (%)					
Yes	24 (49.0)	7 (29.2)	7 (29.2)	10 (41.7)	.011
No	25 (51.0)	0 (0.0)	9 (36.0)	16 (64.0)	

Abbreviations: CBP, Canadian-born parents; FBP, foreign-born parents; IQR, interquartile range; MIRU-VNTR, Mycobacterial Interspersed Repetitive Units–Variable Number Tandem Repeat; SD, standard deviation; TB, tuberculosis.

^a^Percentages have been rounded and may not total to 100%.

^b^Fisher’s exact test (categorical variables) and Kruskal-Wallis rank-sum test (nonnormal continuous data).

^c^Ethnic community is derived from a combination of the region of birth for the pediatric patient and parents of the child.

^d^Excluded “other respiratory” sites, eg, pleura.

^e^Clustered = Yes where the isolate was identical by 24-locus MIRU-VNTR to another patient isolate in British Columbia (2005–2014).

Of the 26 children born outside Canada, 21 (80.8%) were born in Asia (including East, South-Eastern, and South-Central Asia) and the remaining 5 (19.2%) were born in Africa. All were born in high-incidence countries. With respect to ethnic community—defined as a combined measure of the child’s and/or parents’ region of birth—the highest proportion (42.9%) of the 49 pediatric patients were from South-Eastern Asia. Only 7 of 49 children (14.3%) came from a family that had resided in Canada for multiple generations.

Thirty-nine pediatric patients (79.6%) were diagnosed after symptomatic presentation to healthcare providers (Table 1). Six children (12.2%)—all Canadian-born (2 CBP, 4 FBP)—were detected through contact investigations, and 3 (6.1%) foreign-born children were diagnosed as the result of immigration-related postlanding surveillance.

Clinically, 38 (77.6%) children had respiratory involvement (Table 1), and 4 children were characterized as having cavitary disease based on chest radiography (median age: 15.8 years, range: 14.7–17.9). One child in the study was human immunodeficiency virus-positive. Phenotypic drug susceptibilities revealed that 45 (91.8%) isolates were susceptible to all first-line antituberculous medications. Isoniazid monoresistance was seen in 3 individuals (6.1%)—all South-East Asian foreign-born adolescents with Indo-Oceanic lineage strains.

The number of contacts varied considerably between patients, ranging from 0 to 207, with 30 pediatric patients (61.2%) having fewer than 10 contacts (Supplementary Figure 2). Extensive investigations (>50 contacts) were conducted around 3 acid-fast bacilli smear-positive children with respiratory TB. Contact investigation data suggested putative BC-resident source cases for 12 (24.5%) children; in 4 of these instances, the child was diagnosed before the adult source and served as the signal of an active infectious case in the community.

### Molecular and Genomic Epidemiology Investigation of Putative Sources

We first examined the 12 children with putative BC-resident sources identified through contact investigation. In 11 instances, MIRU-VNTR and WGS supported the relationship between the child and assumed source. For the twelfth case, molecular data was not available for the adult TB contact; however, epidemiological evidence strongly corroborated the source of infection. For 8 children (6 Canadian-born [FBP], 1 Canadian-born [CBP], 1 foreign-born), the source was an adult family member regularly residing in the same household as the child. For 4 children (3 Canadian-born [CBP], 1 Canadian-born [FBP]), the source was a visitor to the household who resided elsewhere in BC or Canada. In one of these cases, contact investigation had identified 2 plausible sources—a household member and a visitor; however, the combination of WGS results and epidemiological information suggested the visitor most likely transmitted to both the child and adult household member. One child did not meet either of our definitions for place of acquisition and was classified as having an unknown source.

### Identification of Infections Acquired Out of Province

The MIRU-VNTR revealed that 25 *Mtb* isolates (51.0%) had a unique genotype, suggesting that the infection was likely acquired outside of the province. Indeed, 16 (64.0%) of the children with unique MIRU-VNTR patterns were born in high-burden countries [24]. Of the 9 Canadian-born children with unique MIRU-VNTR patterns, all had foreign-born parents and 7 had a confirmed travel history compatible with acquiring infection overseas. One of the 25 cases was ultimately determined to be the result of transmission in BC from a family member visiting from elsewhere in Canada, leaving 24 cases with an unknown source outside BC.

We also identified 10 cases in which a pediatric *Mtb* isolate shared a MIRU-VNTR genotype with at least 1 other isolate from BC. In 8 cases, the genomic distance between the pediatric case’s isolate and the nearest BC isolate with an identical genotype precluded transmission (81–170 SNVs). In 1 case, the child’s isolate was 6 SNVs away from an *Mtb* isolate from an adult born in the same country as the child and diagnosed in the previous year; however, there was no epidemiological link between the 2, and a distance of 6 SNVs is thought to be incompatible with a transmission event occurring within a single year. In the absence of documented travel history, we concluded that these 9 children had been exposed to TB before emigration. The tenth case represented a foreign-born sibling pair separated by a single SNV, for which epidemiological evidence pointed to infection before emigration from a common source.

**Figure 2.**
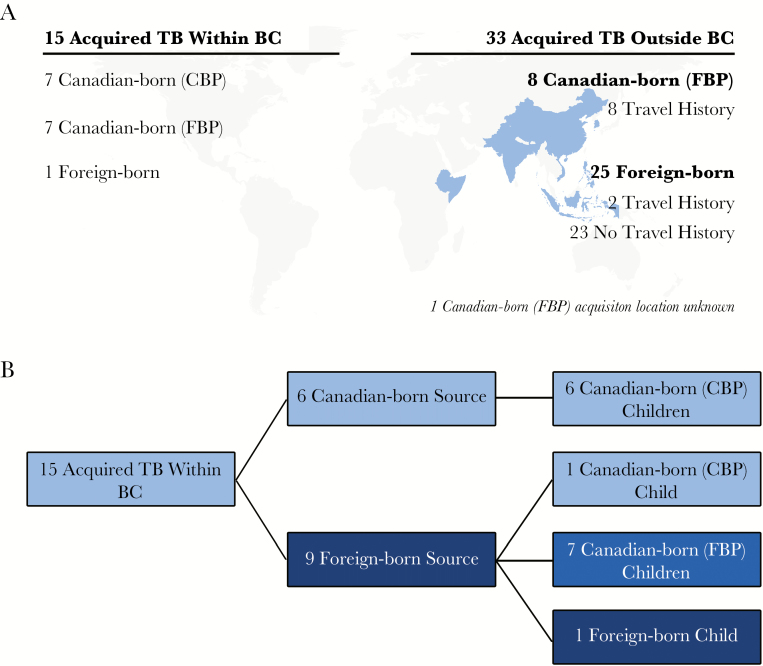
Summary results of molecular epidemiological investigation of culture-positive pediatric tuberculosis (TB) patients, British Columbia (BC), 2005–2014. (A) Summarizes place of acquisition, and countries colored in blue correspond to travel history of patients; (B) stratifies the birthplace of the pediatric patient and source for those in which transmission occurred within BC. Canadian-born parents (CBP); Foreign-born parents (FBP).

Ultimately, whether through MIRU-VNTR or WGS, we found that 33 (67.3%) culture-positive pediatric TB cases diagnosed in BC likely did not arise from local transmission (Figure 2). Epidemiological data suggested that most of these children acquired TB before their arrival in BC (*n* = 23), or through travel to their parents’ birth country (*n* = 8). Two foreign-born children had documented travel histories postarrival to Canada, making it unclear whether their infection was acquired before emigration or was travel-related.

### Identification of Locally Acquired Infections

**Figure 3.**
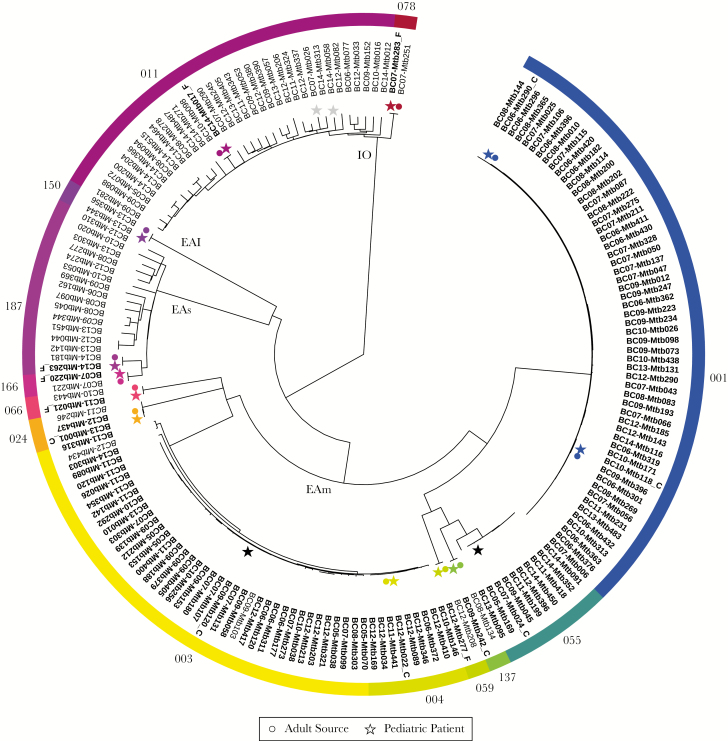
Phylogenetic tree based on whole-genome sequences of *Mycobacterium tuberculosis* isolated from all pediatric diagnoses resulting from whole-genome sequencing (WGS)-confirmed transmission within British Columbia (BC) (*n* = 14), and all adult isolates related by 24-locus Mycobacterial Interspersed Repetitive Units–Variable Number Tandem Repeat (MIRU-VNTR). Genotypic clusters are indicated by colored bands. Pediatric (star) and adult (circle) cases are colored where genomic epidemiology identified a clear source case (*n* = 12). Pediatric cases resulting from community transmission from an unknown source are indicated with black stars (*n* = 2). Gray stars indicate 2 pediatric patients who belong to a MIRU-VNTR cluster frequently seen in BC, for whom WGS indicated their infections were acquired outside BC. Bold tip labels indicate Canadian-born individuals; plain tip labels indicate foreign-born individuals, and italicized tip labels indicate unknown birthplace. Canadian-born children with Canadian-born parents are annotated with “_C” and those with foreign-born parents with “_F”. Internal branches are labeled by lineage: Euro-American (EAm), East-Asian (EAs), EAI (East-Asian Indian), and Indo-Oceanic (IO).

Fifteen (30.6%) culture-positive pediatric TB diagnoses in our study period resulted from presumed local transmission; this includes the 12 cases described earlier, for whom contact investigation suggested a BC resident as the likely source, and 3 additional transmissions identified through WGS, with ≤5 SNVs between the pediatric case and 1 or more adult cases (Figure 3). Of the 15 locally acquired cases, 7 children were born in Canada to CBP. Only 1 WGS-confirmed source was a Canadian-born household family member; instead, 3 sources were Canadian-born visitors to the home, and in 2 cases, although a specific source was not identified, the children were infected with strains known to circulate within their communities. A Canadian-born child (CBP), most likely acquired TB from a foreign-born source (Figure 3) who was not identified through reverse contact investigation. Four of these children belonged to large, previously documented MIRU-VNTR clusters involving largely Canadian-born individuals: MClust-001 (*n* = 56), MClust-003 (*n* = 39), and MClust-055 (*n* = 10) (Figure 3).

Of the remaining 8 patients—7 Canadian-born (FBP) and 1 foreign-born child—WGS (or epidemiology alone [*n* = 1]) suggested infection was acquired within BC from a foreign-born family member; 7 regularly resided in the household. Two Canadian-born children (FBP) belonged to large clusters comprising predominantly foreign-born individuals (MClust-011, MClust-187) (Figure 3).

Our small sample size precluded multivariable regression; however, descriptive statistics (Table 2) indicate that local acquisition was associated with birth in Canada to Canadian parents, age under 5, and infection with the Euro-American *Mtb* lineage.

**Table 2.** Factors Associated With Locally Acquired Tuberculosis, British Columbia, 2005–2014 (*n* = 48)^a^

Characteristic	Acquired Locally		*P* Value^b^
	Yes	No	
Birthplace			
Canadian-born (CBP)	7 (100.0)	0 (0.0)	<.001
Canadian-born (FBP)	7 (46.7)	8 (53.3)	
Foreign-born	1 (3.8)	25 (96.2)	
Age, years			
<5	9 (81.8)	2 (18.2)	<.001
≥5	6 (16.2)	31 (83.8)	
Gender			
Male	7 (28.0)	18 (72.0)	.846
Female	8 (34.8)	15 (65.2)	
Travel History^c^			
Yes	2 (16.7)	10 (83.3)	.292
No	13 (36.1)	23 (63.9)	
Lineage			
Euro-American	10 (83.3)	2 (16.7)	<.001
East-Asian	2 (25.0)	6 (75.0)	
East-African Indian	1 (10.0)	9 (90.0)	
Indo-Oceanic	2 (11.1)	16 (88.9)	

Abbreviations: CBP, Canadian-born parents; FBP, foreign-born parents; TB, tuberculosis.

^a^Source unknown (n = 1).

^b^χ^2^ test (Fisher’s exact test where appropriate).

^c^Travel to a high-incidence TB country.

Of our 49 cases, only 1 remained unclear at the conclusion of our investigation. The Canadian-born (FBP) child had a unique MIRU-VNTR pattern suggesting acquisition overseas, but there was no documented travel history, nor did contact investigation suggest a putative source.

### Household Transmission of Multidrug Resistant-Tuberculosis

Multidrug resistance, defined as resistance to at least isoniazid and rifampin, was observed in 1 pediatric patient with documented exposure to 2 adult TB cases, 1 with MDR-TB, and 1 with a pan-susceptible organism. The MIRU-VNTR genotyping placed this child and both adults into MClust-187; a cluster of cases (*n* = 16) involving an East-Asian lineage strain and predominantly foreign-born individuals with a median age of 66 (IQR, 47–87). Whole-genome sequencing analysis of MClust-187 revealed that the pediatric case was separated from the adult MDR-TB case by a single SNV; the adult contact with the pan-susceptible organism was 197 SNVs apart (Figure 3). Whole-genome sequencing revealed a second transmission pair in MClust-187—a household transmission between family members, both of whom harbored streptomycin-resistant organism—but neither individual was a pediatric case. With distances of 35–247 SNVs between them, the remaining 12 isolates in MClust-187 do not represent local transmission but rather a common region of birth.

## DISCUSSION

In the present study, we used genotyping and genomics to provide the first accurate estimate of TB transmission to pediatric patients in a low-incidence setting, where the majority of all TB diagnoses (73.7%) occur in the foreign-born and are thought to represent reactivation of infections acquired abroad [20]. By coupling a genotyping database including all culture-positive TB isolates diagnosed in BC, Canada (2005–2014) to WGS of clustered isolates and including epidemiological information, we find that one third of culture-confirmed pediatric TB patients acquired their infection within BC. This rate is approximately 3 times that observed when genomics was used to interrogate transmission in a predominantly adult population in a similar low-incidence setting [25], yet it is considerably lower than the pediatric transmission rate we would have estimated based on MIRU-VNTR alone (49.0%).

A lack of laboratory testing and low diagnostic yields in children meant that only half of the notified pediatric cases had an isolate available for genotyping. This limits the present study somewhat, in that we can only reliably assess transmission for cases with a culture-positive specimen. Because novel technologies are making genome sequencing from primary specimens a reality [26], future studies of pediatric cases may be able to examine genomic data from a higher proportion of cases where specimens are submitted. The findings of this study indicate that to more fully understand TB transmission and the molecular epidemiology of a population, culture confirmation should be pursued in all cases.

Our pediatric study TB population assorts into 3 distinct groups. Two thirds of our cases—largely foreign-born older adolescents—likely acquired their infection outside of BC, either before immigration or on a visit to their family’s country of origin. That this group tended to be older teenagers is in line with the findings of an American study reporting differing age distributions between American- and foreign-born children and attributing disease in the latter group to reactivation of latent TB infection (LTBI) [12]. The one third of TB cases likely to have acquired their infection in BC can be further divided into 2 groups: half of these cases were attributed to household transmission from a foreign-born family member, whereas the other half were community transmissions to Canadian-born children from Canadian-born adults, typically in the context of large outbreaks, 2 of which have been described previously [17, 27]. This latter group is notable—studies of pediatric TB cases in other Canadian provinces report 99%–100% of children had foreign-born parents [8, 9], but, in our BC-based study, 1 in 7 children diagnosed with TB had Canadian-born parents. This may reflect differing rates of local transmission between provinces; however, without WGS-based accurate estimates of transmission rates in each province, we cannot confirm this hypothesis.

Although it is often stated that children with active TB serve as a sentinel case indicative of ongoing community transmission, this appears only to be true in particular subpopulations. Here, we only observed community transmission in children of Canadian-born parents, where genomics confirmed that 6 of 7 cases were attributed to community sources. No community transmission was observed in children with foreign-born parents, suggesting that extensive reverse contact investigations may not be warranted in this group. However, it should be noted that we were limited to the detection of active TB infection as a marker of transmission and that not all transmissions result in active disease. Age <5 years is also often associated with local transmission, and, although this was indeed true here, with most children <5 exposed via a household contact, over one third of locally acquired cases were in older children.

We observed that 12 children had travel histories, and in at least 8 of these cases the TB infection was likely acquired while on a trip to their parents’ country of birth. Tuberculosis attributable to travel is often difficult to capture and separate from risk before immigration in foreign-born adults. Significant resources are dedicated to screening immigrants before and upon arrival in Canada; however, in subsequent encounters with the healthcare system, we do not reliably collect travel history for these individuals and tend to attribute their TB diagnoses as LTBI reactivation. Our data indicate that travel to high-incidence settings to visit family poses an infection risk to children, thus it may also contribute to active TB cases among adults who travel to their country of birth. Immigrants traveling to visit friends and relatives in their country of origin are recognized as having increased risks for TB, particularly for long stays [28]. It is interesting to note that at least 3 children in our study who likely acquired their infection during travel visited for less than the 3-month indicator for screening recommended in the Canadian Tuberculosis Standards [19]. Improved education around the risks of travel, better documentation of travel histories, and more aggressive screening protocols may be warranted in individuals returning from high-risk settings involving community-based travel. Our findings (1) are in agreement with other studies regarding the risks of travel for children [8] as well as adults [29, 30] and (2) suggest that new recommendations around screening individuals with community-based travel to high-incidence settings may be warranted.

The retrospective nature of our study meant that we were limited to epidemiological data recorded in our provincial TB registry and, in cases where genomics suggested a source that had not been named in the initial investigation, we were unable to follow up these leads. Furthermore, we could only use molecular data to infer potential source cases diagnosed within the study window, because MIRU-VNTR and WGS data were unavailable for isolates obtained before 2005. This complicates source ascertainment for those children diagnosed in the first few years of our study; however, in each case, the available epidemiological data were sufficient to infer a reasonable source. Prospective WGS of all new culture-positive cases, recently implemented by certain state and national mycobacterial reference laboratories, should allow for more timely and focused contact investigations, particularly in the context of larger outbreaks, where genotyping might suggest many possible sources, and in certain clusters involving foreign-born patients, where a genotypic relationship is infrequently borne out upon WGS.

## CONCLUSIONS

Genomics is changing our understanding of TB transmission dynamics in low-incidence settings, and in the present study, we use its high resolution to more accurately estimate the proportion of pediatric TB attributable to local transmission. Our findings suggest that pediatric TB in BC is a mosaic and that factors including age, place of birth, and travel history must all be considered together when inferring a pediatric patient’s likely exposure. Thus, preventing future pediatric TB cases will likely require a flexible system with varying interventions, in some instances enhanced travel-associated screening, and in others, looking outside the home for source cases. Only through a combination of interventions will we be able to fully address this important issue.

## Supplementary Data

Supplementary materials are available at *The Journal of Infectious Diseases* online. Consisting of data provided by the authors to benefit the reader, the posted materials are not copyedited and are the sole responsibility of the authors, so questions or comments should be addressed to the corresponding author.

Supplementary Material

## Notes

***Acknowledgments.*** We acknowledge the British Columbia Centre for Disease Control (BCCDC) Public Health Laboratory (Tuberculosis [TB]/Mycobacteriology) staff for providing the TB isolates for this study. We also acknowledge Fay Hutton of BCCDC TB Services for her assistance with Integrated Provincial Health Information System data extraction.

***Financial support.*** This work was funded by the BCCDC Foundation for Population and Public Health, the British Columbia Lung Association, the University of British Columbia (4-year doctoral fellowship to J. L. Gu.), the Canadian Institutes of Health Research (doctoral research award to J. L. Gu.), Killam Trusts (doctoral scholarship to J. L. Gu.), the Michael Smith Foundation (scholar awards to J. J. and J. L. Ga.), and the Canada Research Chairs Program (to J. L. Ga.).

***Potential conflicts of interest.*** All authors: No reported conflicts of interest. All authors have submitted the ICMJE Form for Disclosure of Potential Conflicts of Interest.
