# Supplementary material for: Genotyping and Whole-Genome Sequencing to Identify Tuberculosis Transmission to Pediatric Patients in British Columbia, Canada, 2005–2014
Source: J Infect Dis. 2018 May 11;218(7):1155–63. doi: 10.1093/infdis/jiy278 (PMC6107743; doi:10.1093/infdis/jiy278)
Supplement: Supplementary Material [file jiy278_suppl_supplementary.pdf]

SUPPLEMENTARY DATA

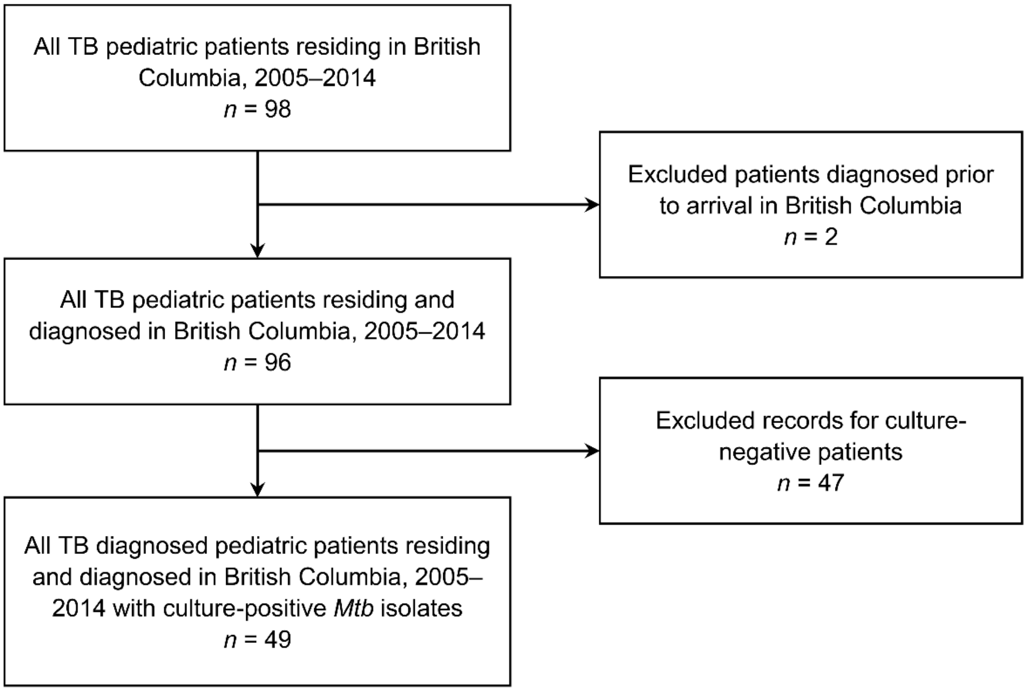

Figure S1. Study inclusion/exclusion criteria. Pediatric was defined as children <18 years of age.

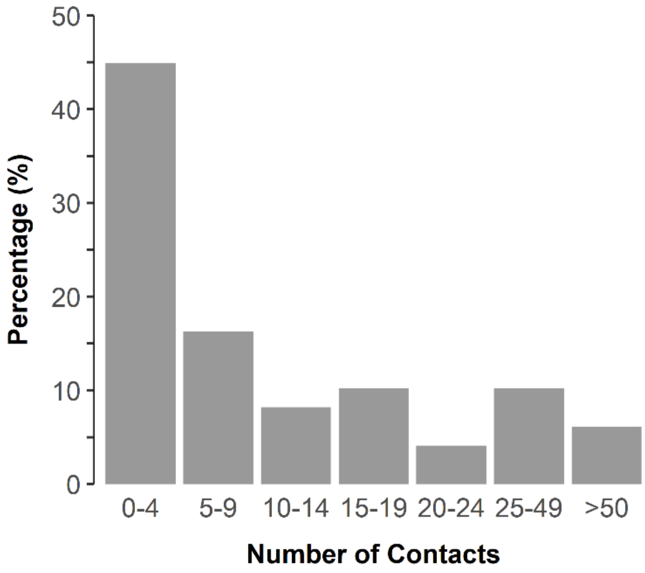

Figure S2. Relative frequency of individuals identified during contact investigation of the pediatric patient.
